# Blood pressure and particulate air pollution in schoolchildren of Lahore, Pakistan

**DOI:** 10.1186/1471-2458-12-378

**Published:** 2012-05-25

**Authors:** Muhammad Sughis, Tim S Nawrot, Syed Ihsan-ul-Haque, Asad Amjad, Benoit Nemery

**Affiliations:** 1 Department of Public Health, Katholieke Universiteit Leuven, Leuven, Belgium; 2 Centre for Environmental Sciences, Hasselt University, Diepenbeek, Belgium; 3 Centre of Research for Public Health, Lahore, Pakistan; 4 Lahore College of Pharmaceutical Sciences, Lahore, Pakistan

## Abstract

**Background:**

Air pollution is a growing health problem for urban populations in emerging economies. The present study examines the (cross-sectional) relation between blood pressure and particulate air pollution in schoolchildren of Lahore (Pakistan).

**Methods:**

We recruited a sample of 8–12 year-old children (mean age 9.9 years; 45% girls) from two schools in Lahore situated in areas with low (n = 79) and high (n = 100) air pollution, respectively. During the study period (January-April 2009) particulate pollution [PM_10_ and PM_2.5_*i.e.* particles with aerodynamic diameters below 10 μm or 2.5 μm, respectively] was measured at the school sites with a laser operated device (Metone Aerocet 531). Blood pressure was measured, after 5 minutes of sitting rest, using an automated device (average of 5 consecutive measurements). Spot urine samples were also collected and concentrations of Na and K were measured.

**Results:**

Mean daily values of PM_2.5_ were 28.5 μg/m^3^ (SD: 10.3) and 183 μg/m^3^ (SD: 30.2), in the low and high pollution areas, respectively. Systolic and diastolic blood pressure were significantly higher in children living in the high pollution area (115.9/70.9 mm Hg) than in the low pollution area (108.3/66.4 mm Hg), independently of age, gender, height, weight, socio-economic status, passive smoking and the urinary concentrations of Na, K, and creatinine.

**Conclusions:**

In 8–12 year-old children, exposure to (traffic-related) air pollution was associated with higher systolic and diastolic blood pressure. These findings, if they persist, might have clinical relevance at older age.

## Background

Numerous epidemiologic studies have indicated that urban air pollution contributes to morbidity and mortality [1,2]. Although air pollution consists of gases (NO_2_, SO_2_, ozone) and particulate matter (PM), the association between air pollution and adverse health effects holds mainly for the particulate fraction, which is expressed as PM_10_ or PM_2.5_*i.e.* particles with aerodynamic diameters below 10 μm or 2.5 μm, respectively. Both short-term peaks in pollution and long-term chronic exposure to PM_10_ or PM_2.5_ have been consistently associated with increased risks of respiratory and, more importantly, cardiovascular disease and death [3-7].

Most evidence on the adverse cardiovascular effects of outdoor air pollution has been obtained from epidemiologic studies in adult and elderly populations [3]. Thus, a cross-sectional study in Los Angeles [8] suggested a role of air pollution in intima-media thickening of the carotid artery and a follow-up study described an association between traffic proximity and the progression of intima-media thickness [9]. Similarly, Hoffmann et al. [5] described more coronary artery calcification in people residing close to heavy traffic than in those living farther away.

In contrast, studies of the effects of air pollution in children have mainly investigated neonatal or infant mortality [10] and respiratory endpoints, such as the incidence of asthma [11] or impaired lung development [12]. Although adult cardiovascular disease, and especially hypertension [13], depends to a large extent on early life circumstances, little research appears to have been done to evaluate cardiovascular parameters, including peripheral blood pressure, in relation to urban pollution in children.

Thanks to legislation, levels of urban air pollution have generally decreased over the course of the latter half of the 20^th^ century in the United States and Western Europe. However, no such trend has taken place in many cities and megacities of developing countries, including Pakistan. According to the World Health Organization, every year, outdoor air pollution accounts for 2 million human lives lost worldwide, with the majority of these deaths occurring in developing countries [14]. Thus, in Asia, measured particulate matter concentrations in many large cities are greater than air quality standards that have been adopted in developed countries [15]. In Pakistan, mean annual PM_10_ ranged from 276 μg/m^3^ in Rawalpindi to 368 μg/m^3^ in Lahore [16]. For comparison, the health-based standard proposed by WHO for annual PM_10_ is 20 μg/m^3^[17].

The aim of our study was to investigate the adverse effects of exposure to air pollution among children from Lahore. In this article, we report on our observations regarding arterial blood pressure in schoolchildren living in two areas of Lahore that differed markedly in exposure to traffic-related air pollution.

## Methods

### Study areas and subject recruitment

**Figure 1:**
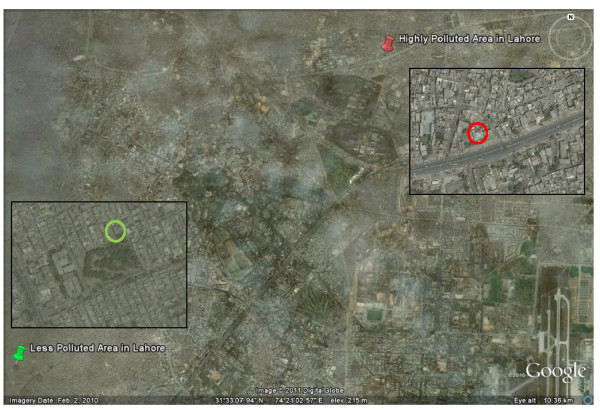
**Google Earth overview of Lahore, Pakistan (accessed on 9 February 2011), with insets (accessed on 10 June 2011) showing the school situated in a low pollution area close to a park (left) and the school situated in a high pollution area close to a major road (right).** The distance between the two schools is ~10 km. All children lived within ~3 km of their school.

Due to the unstable everyday political and security situation, it was difficult to approach various schools; therefore, we used an *ad hoc* strategy of selecting two schools situated in two areas with contrasting exposure to traffic-related air pollution. Our target was to include a maximum number of children between 8 and 12 years in each school. The high pollution school was located next to a highway and the low pollution school was located next to a park in a relatively newly developed residential settlement (Figure 1). The distance between the two schools was ~10 km. Based on the residential addresses and Google Earth; all the children lived in close proximity (within ~3 km) of their school. The children were studied in early 2009: from 12 till 23 January for the school in the high pollution area, and from 1 till 9 April for the school located in the low pollution area. The study was conducted in accordance with the World Medical Association Declaration of Helsinki - Ethical Principles for Medical Research Involving Human Subjects and ethical approval was obtained from Lahore College of Pharmaceutical Sciences. Consent to participate was obtained from the parents and the children themselves; participants were free to withdraw at any time during the study; no dangerous or invasive measurements were performed; confidentiality of the results was guaranteed.

The school officials were first contacted through private contacts. The school officials then communicated with the parents seeking their approval for their children to participate in the study. The children received verbal and written explanation about the process and purpose of the examination and interview. The sole criteria to participate in the study were the prescribed age range, approval of parents and willingness of the participant. All children between 8 and 12 years of age (n = 192) were asked to participate in the study. Of the 192 children, 179 (94%) agreed to participate in the study. Blood pressure could not be measured in 13 children (6 in the low pollution area and 7 in the high pollution area) because their arms were too thin, so the analysis consists of 166 participants. Several endpoints were investigated, including spirometry and exhaled NO, but this article will deal with BP and related data.

### Exposure to PM and environmental noise

We used a portable laser-operated aerosol mass analyzer (Aerocet 531, Met One Instruments Inc, USA) to measure PM and humidity. This instrument measures PM_1_, PM_2.5_ and PM_10_ mass concentrations in μg/m^3^ and had been previously calibrated against a European monitoring station (Flemish Environment Agency, Borgerhout, Antwerp, Belgium). Measurements of PM were obtained at the study sites (outdoors, but under a roof of the school playground) for at least 24 h before each examination day; for each subject, the average PM_1_, PM_2.5_ and PM_10_ values were calculated for the previous 24 h and varied slightly between subjects examined on the same day, with outdoor sampling periods of 2 min being taken at 10 min intervals. In addition, measurements of PM were also taken indoors for 10 consecutive sampling periods of 2 min (20 min in total) between 10 am and 11 am on the examination days. Ambient temperature and relative humidity were measured simultaneously. Because, high humidity tends to give wrong PM values with the Aerocet, the values of PM obtained when relative humidity was above 80% were excluded. Minimum and maximum sound levels were measured using a sound level meter (Model 407730 Extech Instruments Corporation, USA) between 10 and 11 am on the examination days.

### Questionnaire and anthropometric measures

The sampling campaign was organized during the school time (9 am and 1 pm) and 10 children per day were examined. To assess lifestyle, use of tobacco (active and passive), intake of medicines, exposure to biomass cooking and social class of parents, we used a pre-tested questionnaire adapted from the questionnaire of the International Study of Asthma and Allergies in Childhood [18] administered in Urdu (the national language of Pakistan). The questionnaire was administered in an interview face-to-face by trained staff. The interview was conducted within the school premises in school time and lasted for approximately 5–7 min. Social class was estimated on the basis of paternal education and was defined as low (8 years of education or less), middle (8 to 12 years) and high (12 to 16 years or more). Age was calculated as full years since date of birth (n = 173) or as given by parent/guardian (n = 6). Standing height was measured without shoes with flat foot touching the wall. Weight was measured only with necessary clothing and without shoes and rounded off to the nearest kg. Body mass index (BMI) was calculated by dividing weight by squared height in meters.

### Clinical measures

After the subjects had rested for 5 min in the sitting position, we measured blood pressure by making five consecutive readings using an automated blood pressure instrument (Stabilo Graph, Germany) with a standard cuff suitable for <33 cm arm circumference. The guidelines of the European Society of Hypertension were followed for the measurement of blood pressure [19]. The mean of the five measurements was used for analysis and also to classify participants into one of the Joint National Committee (JNC) VII on blood pressure categories: normal blood pressure (systolic <120 mmHg and diastolic <80 mmHg), pre-hypertension blood pressure (systolic 120–139 mmHg or diastolic 80–89 mmHg) [20]. If systolic and diastolic blood pressure readings belonged to different categories, the higher of the two readings was used to determine the blood pressure category.

A spot urine sample was collected in the end, after the questionnaire, anthropometric and clinical measurements, and analyzed for creatinine on the same day, using a kit from DiaSys Diagnostic Systems (Germany) as per instructions from the manufacturer. The remaining sample was stored in a domestic freezer and later shipped to Leuven, Belgium for further analysis. In the urine sample the concentrations of sodium (Na) and potassium (K) were analyzed using an automated analyzer (Modular P800-ISE900 System, Roche Diagnostics; Mannheim, Germany) at the Clinical Laboratory of Ziekenhuis Oost-Limburg, Genk, Belgium (Dr. J. Penders).

### Statistical analysis

We used SAS software version 9.2 (SAS Institute Inc, Cary, NC). For comparison of means, medians and proportions we applied Student’s *t*-test, Wilcoxon’s test and the chi-square statistics, respectively. The other statistical techniques included general linear models PROC GLM and logistic regression. We applied logistic regression analysis to study the association between pre-hypertension, area of residence and particulate air pollution. Models were constructed based on parameters known to be biologically associated with blood pressure including urinary sodium, potassium, height, weight, age and gender. We considered height instead of body mass index in our adjusted models as this variable is more closely linked with blood pressure in children [21,22]. P values below 0.05 (two-sided) were considered statistically significant.

## Results

The characteristics of the 166 children and the measured PM levels are listed in Table 1. As anticipated from the location of the schools with respect to traffic, the average values of PM measured in the 24 h before examinations were markedly higher in the high pollution area [mean PM_2.5_ 183 μg/m^3^] than in the low pollution area [mean PM_2.5_ 28.5 μg/m^3^]. Mean outdoor temperature did not differ between the two schools (15.5°C vs 15.0°C), but relative humidity did differ somewhat (58% vs 67%) due to more rainy days during the first period of study. On the days (n = 4) when humidity was above 80%, the PM data was excluded from analyses. PM values were lower on rainy days. Values of indoor PM were significantly (paired *t*-test) lower than those measured outdoors in the high pollution area (p < 0.0001), but not in the low pollution area. None of the participants’ families used biomass for cooking or heating. Environmental noise measurements gave mean (SD) values of 44 (0.9) dB(A) and 72 (1.1) dB(A) in the low pollution school *versus* 52 (10.7) dB(A) and 84 (2.7) dB(A) in the high pollution school.

**Table 1:** Characteristics of children attending school in a low and a high pollution urban area

Characteristics	Low Pollution School (n=73)	High Pollution School **(**n=93)	P-value
Male sex - no. (%)	46 (63%)	46 (49.4%)	
Age - yr	10.0 (8.0-11.0)	10.0 (9.0-11.0)	0.2
Height - cm	134 (125–143)	134 (130–138) 0.9	0.9
Weight - kg	27.0 (22.0-34.0)	27.0 (25.0-31.0)	0.9
BMI - kg/m^2^	15.3 (14.1-16.4)	15.3 (14.4-17.1)	0.9
Passive smoking	22 (30%)	34 (36%)	0.3
Socio-economic class§			0.001
Low	7 (10%)	31 (34%)	
Middle	24 (33%)	22 (24%)	
High	41 (57%)	39 (42%)	
Outdoor PM_1_ - μg/m^3^	7.3 (5.3)	58.2 (9.6)	<0.0001
Outdoor PM_2.5_ - μg/m^3^	28.5 (10.3)	183.0 (30.2)	<0.0001
Outdoor PM_10_ - μg/m^3^	223.0 (93.5)	728.6 (53.5)	<0.0001
Indoor PM_1_ - μg/m^3^	8.4 (5.5)	52.7 (12.1)	<0.0001
Indoor PM_2.5_ - μg/m^3^	29.1 (15.1)	163.0 (61.6)	<0.0001
Indoor PM_10_ - μg/m^3^	222.9 (119.4)	590.7 (219.4)	<0.0001
Outdoor temperature - °C	15.5 (3.3)	14.9 (4.5)	0.4
Relative humidity - %	58.4 (16.4)	67.1 (18.5)	0.001

The data is mentioned as median (25^th^ - 75^th^ percentile), number (%), or mean (standard deviation) § according to paternal educational level (no information for one participant in each group). Particulate Matter (PM) was measured continuously with Aerocet 531 device during the previous 24 hours (outdoor) or between 10 and 11 am on the day of examination (indoor); corresponding averages were calculated for the different fractions, *i.e.* particles with diameter below 1 μm (PM_1_), 2.5 μm (PM_2.5_) and 10 μm (PM_10_). Values of PM were excluded when humidity was > 80% (4 days, high pollution school).

The age, gender, height, weight and BMI distributions did not differ between children from the two schools (Table 1); however, the parents of the children from the low pollution area were significantly more educated (and hence presumably wealthier) than those from the high pollution area. In the whole group, systolic and diastolic blood pressure averaged (SD) 115.6 mmHg (SD: 8.7) and 70.6 mmHg (SD: 7.6), respectively. Multiple regression analysis revealed a higher systolic blood pressure in girls compared with boys [(regression coefficient ± SE) +3.1 ±1.64 mmHg; p = 0.05], and an increase of systolic blood pressure with weight (+0.55 ± 0.15 mmHg per kg; p = 0.0005], but no association with age (p = 0.19), socio-economic status (p = 0.76), height (p = 0.91), urinary Na (p = 0.97), K (p = 0.99), temperature (p = 0.21), humidity (p = 0.12), passive smoking (p = 0.69) and creatinine (p = 0.67). For diastolic blood pressure, girls tended to have a higher value (+2.2 ± 1.49 mmHg; p = 0.13) than boys, while none of the other studied determinants including age (p = 0.09), socio-economic status (p = 0.05), weight (p = 0.69), height (p = 0.21), urinary Na (p = 0.39), K (p = 0.86), temperature (p = 0.71), humidity (p = 0.64), passive smoking (p = 0.97) or creatinine (p = 0.25) were significant. Nevertheless, independently of the level of significance, these variables, considered as classical determinants of blood pressure (*i.e.*, gender, age, socio-economic status, weight, height, urinary Na, and K) were forced into all adjusted models.

**Figure 2:**
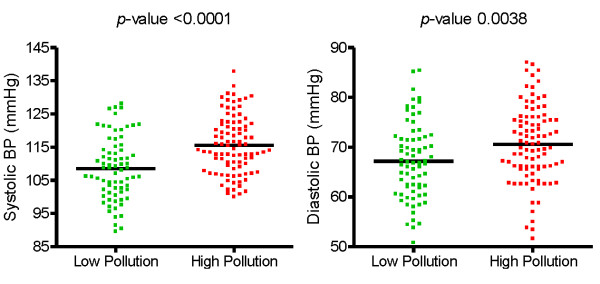
Difference between systolic (left) and diastolic (right) blood pressure among schoolchildren living in a low pollution (n = 73) vs high pollution (n = 93) area in Lahore.

Values of systolic and diastolic blood pressure were significantly higher in the children living in the highly polluted area than in those from the less polluted area (Figure 2). These differences remained when values of systolic and diastolic blood pressure were adjusted for gender, age, socio-economic status, weight, height, Na, K, passive smoking and creatinine. Heart rate was higher by 3 bpm in the children from the less polluted area (Table 2).

**Table 2:** Blood pressure and urinary measurements of children in low and high pollution urban area

Characteristics	Low Pollution School (n=73)	High Pollution School (n=93)	P-value
Systolic blood pressure - mmHg*	108.3 (106.1 to 110.6)	115.9 (114.0 to 117.9)	<0.0001
Diastolic blood pressure - mmHg*	66.4 (64.4 to 68.4)	70.9 (69.2 to 72.7)	0.002
Pulse - bpm*	98.1 (94.8 to 101.4)	94.9 (92.1 to 97.7)	0.2
Urinary creatinine - g/l	0.7 (0.2)	1.3 (0.8)	<0.0001
Urinary Na - nmol/l**	127.1 (109.5 to 147.5)	131.5 (115.3 to 149.2)	0.7
Urinary K - nmol/l**	54.8 (46.1 to 65.2)	45.1 (39.0 to 52.2)	0.1

The data is mentioned as mean (standard deviation) for creatinine and as adjusted mean (95% CI) with adjustment made for gender, age, height, weight, urinary sodium, potassium, socio-economic status, passive smoking and creatinine (*), or adjusted mean (95% CI) with adjustment made for gender, age, height, weight, socio-economic status and creatinine (**).

**Figure 3:**
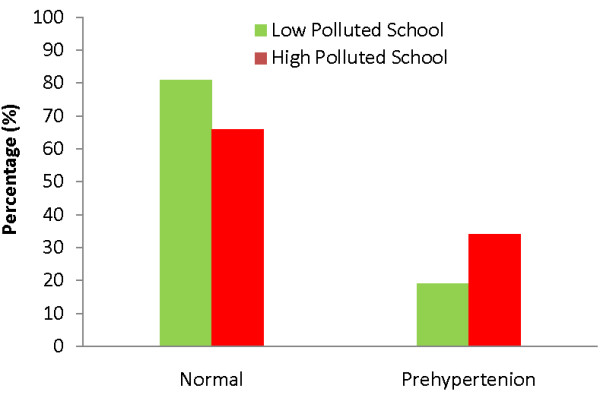
**Blood pressure distribution among school children living in high pollution area vs a less polluted area.** Normal blood pressure (systolic <120 mmHg and diastolic <80 mmHg) and prehypertension (systolic 120–139 mmHg or diastolic 80–89 mmHg) are defined according to VII Joint National Committee on Prevention, Detection, Evaluation, and Treatment of High Blood Pressure [20], (p=0.03).

When we applied the criteria of JNC VII blood pressure categories [20], 59 (81%) children living in the low pollution area and 61 (66%) children living in the high pollution area had normal blood pressure; 14 (19%) subjects living in the less polluted area and 32 (34%) subjects living in the more polluted area had blood pressure in the pre-hypertensive range (Figure 3). None of the children had stage 1 hypertension (systolic 140–159 mmHg or diastolic 90–99 mmHg).

Logistic regression, with adjustments as before, revealed an odds ratio for not having a normal blood pressure of 2.56 (95% CI: 0.96 to 6.78; p = 0.058) for those living in the area with high PM exposure compared with those living in the area with low PM.

## Discussion

In this cross-sectional study we observed that children attending a primary school situated in an area of high air pollution, close to heavy traffic, had a substantially higher arterial blood pressure than children attending a school situated in an area with less air pollution. Our findings are novel on at least two accounts: to our knowledge, our study is the first to investigate the adverse health effects of urban air pollution in children from Pakistan and, moreover, this is one of the first studies to report an association between particulate air pollution and blood pressure in children. With mean differences of 7.6 mmHg for systolic blood pressure and 4.5 mmHg for diastolic blood pressure, the differences between the two groups of children are not only statistically significant, they are also substantial and, therefore, likely to be biologically relevant with regard to the long-term risk of cerebrovascular disease and coronary events.

The differences in blood pressure between the two groups are unlikely to be due to technical factors. Blood pressure was measured according to guidelines of the European Society of Hypertension [19] using a technically validated automated device, thus eliminating any investigator-dependent bias [23]. All measurements were made in similar, standardized circumstances, over a period of 4 months (January to April 2009), with the children having been sitting for at least 5 minutes, and with 5 consecutive readings being obtained.

In the absence of technical factors or measurement errors that could explain the differences in blood pressure between the two groups, the following factors can be invoked to explain why the children from the most polluted environment had a higher blood pressure: dietary differences (with a higher salt intake), higher exposure to traffic-related noise and air pollution.

The dietary intake of sodium is a well-established determinant of blood pressure in adults and children [24]. The two groups of schoolchildren differed in their socio-economic status (as assessed by father’s educational level) and it is, therefore, conceivable that they also differed with respect to their diet, either qualitatively or quantitatively, and so we have adjusted for socio- economic status as a confounder in our analyses. However, we do not believe these differences in socio-economic status to be very important. First, the two groups of schoolchildren did not differ with regard to height and weight, thus excluding any large quantitative differences in food intake. Second, the two groups did not differ significantly with regard to their urinary sodium concentrations, thus suggesting similar intakes of salt for both groups. The values of creatinine did differ considerably between the two groups, with lower values (mean of 0.76 g/l) being found among the low pollution group and higher values (mean 1.32 g/l) among the high pollution group. We have no explanation for this finding. Technical factors do not seem to be a reason for this finding, because creatinine concentrations were reassessed in Belgium in a random 10% of the samples and found to be similar as the ones measured locally (data not shown). There was undoubtedly more noise at the school exposed to high pollution than at the less polluted school. Minimum and maximum sound levels were indeed higher in the high pollution school than in the low pollution school, but this does not mean that the average noise level was higher by 8 to 12 dB (A) in the high pollution school compared to the low pollution school, because the instrument used did not allow us to assess time-weighted average noise levels. It was, therefore, impossible to correct for any effect of exposure to noise during the measurements of blood pressure. Moreover, as suggested by various investigators [25,26], to the extent that traffic causes both noise and air pollution, it is difficult to disentangle the effects of these two factors. Moreover, the results of studies on the effects of (road and aircraft) noise have been somewhat contradictory [27]. In a recent study of 1048 German schoolchildren, children living in busy traffic streets had only slightly higher values of systolic blood pressure (1.8 mmHg, p < 0.05) and diastolic blood pressure (1.0 mmHg, NS) than those living in low traffic streets [28].

In preschool children from Belgrade, a higher difference in systolic blood pressure (5 mmHg) was found in children from noisy residences compared to those from quiet residences [29]. So, we cannot exclude that differences in exposure to traffic noise were partly responsible for the differences in blood pressure between our two groups of children.

In view of the established effects of particulate pollution on the cardiovascular system, we must consider traffic-related air pollution by particulate matter as a plausible explanation too. In studies of air pollution, it is important to distinguish short-term temporal effects of peaks of air pollution - which act as triggers of adverse events such as myocardial infarction [30] - from the chronic effects of continuous exposure to air pollution. Although we did measure daily PM levels in our study, and although these varied somewhat from day to day during the course of the study, there were no significant relations within each group between blood pressure and current PM or PM over the past 24 hours (data not shown). These relations were, however, significant when we considered the entire group, but this was dominated by the spatial contrast in PM between the two groups, because the effects of PM remained when we took temporal variability into account in the analysis. Consequently, we attribute the difference in blood pressure between the two groups of schoolchildren to the difference in their chronic exposure to air pollution.

Many epidemiological studies have found a consistent relation between long-term exposure to urban particulate exposure and morbidity or mortality from cardiovascular disease in adults, and experimental studies provide mechanistic plausibility for these associations [3]. Several recent studies have also found proximity to heavy traffic to be more closely associated with adverse health effects than estimated exposure to particulate matter [1,3,5,9,12,31,32]. Very few studies have reported on cardiovascular effects of proximity to traffic in children. Iannuzzi et al. [33] found a statistically significant difference in carotid stiffness, but not in carotid intima-media thickness or peripheral artery pressure, among 52 children according to their estimates exposure to air pollution. Calderón-Garcidueñas et al. [34] attributed to air pollution the higher pulmonary arterial pressure found in children living in Mexico City, compared to control children exposed to lower levels of PM_2.5_, but they did not report on arterial blood pressure in these children. Recently, a large epidemiologic study showed that preschool children exposed to environmental tobacco smoke had on average 1 mmHg higher systolic blood pressure than children not exposed to environmental tobacco smoke [35]. As with polluted ambient air, environmental tobacco smoke is largely composed of an aerosol of particles derived from combustion [36], therefore, our conclusions for outdoor air pollution and those for passive smoking mutually support each other.

What are the possible implications of our observations that children living and attending school in an environment with higher levels of air pollution, presumably as a result of traffic, have a higher arterial blood pressure than children living in a less polluted environment? Although no child had hypertension and although the mean systolic blood pressure in the most exposed group was below 140 mmHg, mean differences of about 7 mmHg for systolic blood pressure and about 4 mmHg for diastolic blood pressure are substantial. Thus, in our population, the odds of having a blood pressure above “normal” blood pressure (systolic <120 mmHg and diastolic <80 mm Hg) was 2.56 times higher in those living in the highly polluted area. Admittedly, the criteria used here for defining normal blood pressure and pre-hypertension are those applicable for adults from industrially developed countries [20]. Although it remains to be determined whether the same limits apply for the children studied here, we have used these categories to put our results into some clinical perspective. At the population level, pre-hypertension (systolic 120–139 mmHg or diastolic 80-89 mmHg) is associated, in adults, with a higher incidence of hypertension [37] and an increased risk of cardiovascular complications [38] compared with normal blood pressure. Risk factors in children may persist into later life and eventually lead to cardiovascular disease [39]. Findings from the Bogalusa Heart Study in the USA demonstrated that childhood blood pressure levels at or above the 80^th^ percentile, *i.e.* not necessarily in hypertensive ranges, were associated with an increased prevalence of elevated blood pressure during adulthood [39]. A follow-up study [13] showed that blood pressure in a group of male students at the age of 20.5 years was associated with the incidence of cardiovascular diseases in the following 41.3 years. These findings indicate that elevated blood pressure during young age may have later clinical significance.

In the absence of similar studies from other areas, we do not know how representative our findings are for other populations. It has to be noted that in the present study, the outdoor PM levels measured in the high pollution area were extremely high (average PM_10_ above 700 μg/m^3^, average PM_2.5_ above 180 μg/m^3^) and still high, by Western standards, even in the “less polluted environment” (average PM_10_ above 220 μg/m^3^, average PM_2.5_ around 29 μg/m^3^). These relatively low proportions (15-25%) of PM_2.5_ to PM_10_ indicate that in Lahore - as in other locations from developing and emerging countries [15] - the high PM levels are largely due to coarse particulates, *i.e.* presumably resuspended dust.

Our study has limitations including the cross-sectional nature. Thus the blood pressure measurements were taken on just one day, which did not account for inter-day variability. We did not measure gases (NO_2_ and O_3_), nor residential PM. Also, personal exposure to particulate air pollution was not recorded. Due to the aggregation of location and air pollution, we could not control for location in the statistical models. Another limitation is that we only studied a limited number of children from just two schools, so we do not know how representative the results are for the whole city of Lahore, let alone other cities. However, the results of our study justify a larger study on the effect of PM on blood pressure in children.

## Conclusions

In conclusion, we discovered that children living and attending school in an area of very high traffic-related air pollution had a substantially higher arterial blood pressure compared to children with less exposure. Although we are aware of the limitations of a cross-sectional study with regard to causality and prognosis, we conclude that traffic-related urban pollution may contribute, even in children, to their later risk of hypertension and cardiovascular complications, if this higher blood pressure persists.

## Competing interests

We declare that we have no conflict of interests.

## Authors’ contributions

MS, TSN, and BN designed the study. MS collected data. SI and AA assisted MS in the data collection. MS, TSN, and BN contributed in the discussion of data, drawn conclusions, drafted the manuscript, and approved the final manuscript. All authors read and approved the final manuscript.

## Pre-publication history

The pre-publication history for this paper can be accessed here:

http://www.biomedcentral.com/1471-2458/12/378/prepub
